# Roles of microRNAs in atherosclerosis and restenosis

**DOI:** 10.1186/1423-0127-19-79

**Published:** 2012-08-29

**Authors:** Li-Jing Chen, Seh Hong Lim, Yi-Ting Yeh, Sheng-Chieh Lien, Jeng-Jiann Chiu

**Affiliations:** 1 Division of Medical Engineering Research, National Health Research Institutes, Miaoli, 350, Taiwan; 2 Institute of Molecular Medicine, National Tsing Hua University, Hsinchu, 300, Taiwan; 3 Institute of Bioinformatics and Structural Biology, National Tsing Hua University, Hsinchu, 300, Taiwan; 4 Institute of Molecular & Cellular Biology, National Tsing Hua University, Hsinchu, 300, Taiwan

## Abstract

Atherosclerosis is commonly appreciated to represent a chronic inflammatory response of the vascular wall, and its complications cause high mortality in patients. Angioplasty with stent replacement is commonly performed in patients with atherosclerotic disease. However, the restenosis usually has a high incidence rate in angioplasty patients. Although the pathophysiological mechanisms underlying atherosclerosis and restenosis have been well established, new signaling molecules that control the progress of these pathologies have continuously been discovered. MicroRNAs (miRs) have recently emerged as a novel class of gene regulators that work via transcriptional degradation and translational inhibition or activation. Over 30% of genes in the cell can be directly regulated by miRs. Thus, miRs are recognized as crucial regulators in normal development, physiology and pathogenesis. AIterations of miR expression profiles have been revealed in diverse vascular diseases. A variety of functions of vascular cells, such as cell differentiation, contraction, migration, proliferation and inflammation that are involved in angiogenesis, neointimal formation and lipid metabolism underlying various vascular diseases, have been found to be regulated by miRs. This review summarizes current research progress and knowledge on the roles of miRs in regulating vascular cell function in atherosclerosis and restenosis. These discoveries are expected to present opportunities for clinical diagnostic and therapeutic approaches in vascular diseases resulting from atherosclerosis and restenosis.

## Review

### Introduction

Atherosclerosis is a chronic and progressive pathology characterized by the accumulation of lipid and fibrous elements in the large arteries, which causes a number of cardiovascular-related diseases. Atherosclerosis has a tremendous impact in developing and developed countries, representing the underlying cause of approximately 50% of deaths. Our knowledge of the pathophysiology for this important malady has evolved over the past century. Extensive evidence reveals that the pathogenic feature of atherosclerosis is an inflammatory process, in which vascular endothelial cells (ECs) become dysfunctional due to influences by chemical substances, such as cytokines and growth factors [1], and hemodynamic forces [2]. Activated ECs with high expression levels of various leukocyte adhesion molecules recruit leukocytes and monocytes to bind to the endothelium and migrate into the vessel wall. The lesion then experiences the following steps: foam cell formation, fatty streak accumulation, migration and proliferation of vascular smooth muscle cells (VSMCs), and fibrous cap formation. Finally, the rupture of the unstable fibrous cap causes thrombosis in complications of advanced lesions that lead to unstable coronary syndromes, myocardial infarction and stroke. The knowledge that atherosclerosis is a vascular pathology resulting from inflammatory response enables new approaches to treatment and prevention. Immunosuppressant and anti-inflammatory agents could potentially be applied in clinical trials. However, surgical treatments remain the prevalent method of treating in the patients with atherosclerosis, including percutaneous transluminal coronary angioplasty (PTCA) and stent placement.

Angioplasty and stent placement remove the occlusion to augment the inner diameter of the artery at various vascular locations. These treatments exclusively improve the hemodynamic flow rate and lead to normal blood flow. While these treatments have been used in many patients with atherosclerotic disease over the past decades, restenosis is an ongoing complication with an incidence of 30–40% within 3–6 months after treatment. Although restenosis and atherosclerosis are recognized as inflammatory processes in response to injury [3], restenosis is in fact a vascular injury caused by balloon dilation and stent replacement during angioplasty [4]. The development of restenosis is pathophysiologically distinct from atherosclerosis. These differences have been observed during the proliferation and migration of VSMCs, extracellular matrix remodeling and neointimal hyperplasia. Anatomic and procedural clinical variables are associated with an increased incidence of restenosis following angioplasty [5].

MicroRNAs (miRs) are recently emerging endogenous, noncoding, single-stranded RNAs of 18–22 nucleotides that constitute a novel class of gene regulators. The first miR, lin-4, was discovered during the development of *Caenorhabditis elegans* in 1993 [6]. Bentwich et al. [7] developed an integrative approach combining bioinformatic prediction with microarray analysis and sequence-directed cloning to reveal that more than 800 miRs exist in humans. Currently, over 15,000 miR gene loci have been identified in over 140 species, and more than 17,000 distinct mature miR sequences are present in the miRBase16 [8]. MiRs bind to their target genes in 3’-untranslated regions (3’-UTRs), leading to the direct degradation of messenger RNA (mRNA) or translational repression by a perfect or imperfect complement. This implies that miRs are able to regulate the expression of hundreds or thousands genes. Thus, it is not surprising that miRs are involved in the regulation of all major cellular functions [9].

The pathophysiological mechanisms of vascular pathologies such as atherosclerosis, hypertension, coronary artery disease and restenosis after angioplasty have been well established over the past decades. Vascular properties including angiogenesis, re-endothelialization and neointima formation contribute to these vascular pathologies/diseases. Inflammatory responses to injury, differentiation, proliferation, migration and apoptosis of VSMCs or ECs are critical cellular events for the development of these vascular diseases. Blood cell recruitment, infiltration, activation and differentiation are also involved in these complicated diseases. Vascular diseases have been widely explored, and many new molecules have been studied as potential clinical therapies. In recent years, the roles of miRs have gradually received increasing attention in the biology of vascular diseases. Altered miR expression profiles have been related to cardiovascular diseases in over 400 studies. Although several review articles have described the regulation of miRs in vascular remodeling, inflammation and diseases [10-12], the specific role of miRs in the regulation of atherosclerosis and restenosis is barely described. Hence, this review focuses on the roles of miRs in different types of vascular cells in relation to atherosclerosis and restenosis.

### The biogenesis of microRNA

#### Primary miR

**Figure 1:**
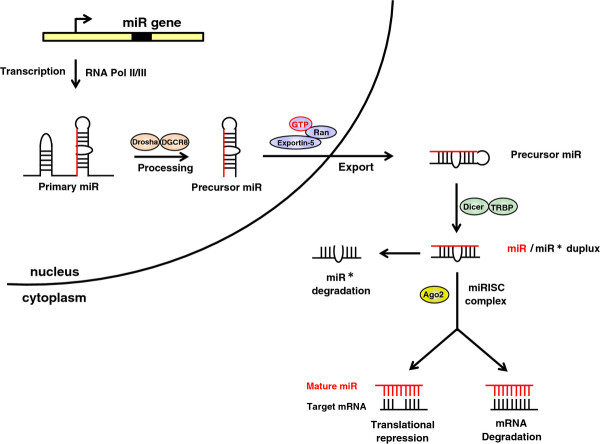
**The canonical pathway of miR processing.** The primary miR (pri-miR) are transcripted by either RNA polymerase II or III from independent gene in the nucleus. In the following processing, the microprocessor complex (Drosha-DGCR8) processes pri-miR into a ~60-100-nucleotide precursor hairpin (pre-miR). The resulting pre-miR is exported to the cytoplasm by Exportin-5-Ran-GTP. In the cytoplasm, the RNase III Dicer and TRBP cleave the pre-miR into ~22-nucleotide miR/miR* duplex. One strand termed as guide strand, further representing a mature miR, the miR* termed as passenger strand, which undergoes degradation rapidly. Mature miR is incorporated into a miRISC and base-paired to its target mRNAs for mRNA degradation or translational repression.

Most miR genes are located in intronic regions, which may be transcribed as part of the mRNA genes. As for general mRNA, miR genes are commonly transcribed by RNA polymerase II (pol II) [13] in the nucleus (Figure 1). The primary miR transcripts (pri-miRs) contain capped structures and polyadenylated (poly A) tails, the hallmark properties of class II gene transcripts [14]. Besides pol II, Borchert *et al*. [15] found that C19MC miRs, including miR-515-1, miR-517a, miR-517c and miR-519a-1, are expressed by RNA polymerase III (pol III). Some miRs contain primary transcripts to produce a single miR, whereas other transcripts encode proteins in their exons and miRs. The cluster miRs such as the miR-17~92 family are grouped together in one cluster on a single unprocessed transcript and expressed together.

#### Precursor miR

Following transcription by pol II or pol III, the pri-miR received is endonucleolytically cleaved to an ~60–100 nucleotide hairpin structure with an ~2 nucleotide 3’ overhang termed the precursor-miR (pre-miR) by the nuclear microprocessor complex. This microprocessor complex is formed by the RNase III enzyme Drosha (RNASEN) and its partner DGCR8 (DiGeorge critical region 8), also known as Pasha (Partner of Drosha) in *D. melanogaster* and *C. elegans*[16-18]. Several molecules were identified to be involved in the post-transcriptional modulation of miR processing [19]. For example, RNA helicases p68 and p72, the cofactors of the microprocessor complex, promote the Drosha cleavage of a subset of miRs. p53, an important tumor suppressor protein, is present in the complex with p68 and Drosha to enhance the Drosha processing of a subset of miRs. Smad, transforming growth factor (TGF)-β and bone morphogenetic protein (BMP)-specific signaling transducer proteins are recruited to a consensus sequence (R-SBE) within the stem region of the primary transcripts of TGF-β/BMP-miRs in the Drosha and p68 complex. Thus, this Smad-Drosha-p68 complex promotes the processing of TGF-β/BMP-miRs [20]. After nuclear processing, the pre-miR is exported into the cytoplasm by Exportin-5 (XPO5) in complex with Ran-GTP cofactor [21].

#### Mature miR

The pre-miR is further processed in the cytoplasm by another RNase III Dicer, which forms the RISC complex with Argonaute 2 (Ago2) and TRBP (Tar RNA binding protein), which cleaves off the hairpin loop of the pre-miR to generate an ~22-nucleotide miR duplex [22-24]. This miR duplex contains mature miR referred to as the guide strand and a complementary strand referred to as the passenger strand (miR*). Following processing, one strand of the miR/miR* duplex (usually the guide strand) is preferentially incorporated into a miR-induced silencing complex (miRISC) that contains Dicer and other associated proteins [25], whereas the miR* is released and rapidly degraded. As part of the miRISC, the miR is base-paired to its target mRNA to induce translational repression or direct degradation [26,27].

### Atherosclerosis

A growing body of studies reveals that the pathogenic feature of atherosclerosis is an inflammatory process involving ECs in response to injury. These dysfunctional ECs lead to a sequence of inflammatory responses, blood-cell accumulation, foam cell formation, fibrous formation, advanced plaque formation and rupture [1,28,29]. These complicated processes are contributed by various blood cells such as monocytes, macrophages, and lymphocytes, and vascular cells such as ECs and VSMCs. Moreover, these cells influence each other and secrete various cytokines and growth factors to promote the formation of atherosclerosis.

#### Initiation step

The endothelium consists of a single layer of vascular ECs and serves as a selective barrier between blood and tissues. Atherosclerotic plaques preferentially occur in specific arterial sites such as branches, bifurcations and curvatures in which the flow pattern is disturbed, with a lower velocity and no particular orientation. ECs tend to turn over in these regions and show increased permeability to macromolecules such as low-density lipoprotein (LDL). As a result, LDL diffuses passively through EC junctions and accumulates in the subendothelial matrix. Subsequently, the LDL undergoes modification and oxidation, contributing to inflammation and further foam cell formation.

#### Inflammation

In the initial lesion, ECs have an activated and pro-inflammatory phenotype that leads to expression of various EC adhesion molecules (such as intercellular adhesion molecule-1 [ICAM-1], vascular cell adhesion molecule-1 [VCAM-1] and E-selectin), growth factors such as macrophage colony stimulating factor (M-CSF), and chemokines including monocyte chemotactic factor-1 (MCP-1) [30]. E-selectin is a member of the selectin family of adhesion molecules that plays a crucial role in the initial interaction between circulating leukocytes and ECs. E-selectin binds to carbohydrate ligands on the leukocytes and facilitates the rolling of leukocytes along the endothelial surface. Under the cooperation of adhesion molecules and chemotactic factors, the rolling leukocytes enter the vessel wall. In addition, the circulating monocytes and lymphocytes are recruited by MCP-1 and M-CSF into the vessel wall. M-CSF promotes macrophage proliferation and differentiation and the expression of scavenger receptors (SR), which increases the production of cytokines and growth factors by these cells. LDL has to be modified and oxidized before it can be taken up by macrophages. The reactive oxygen species (ROS) produced by vascular cells, including sphingomyelinase, secretory phospholipase-2 (sPLA2) and myeloperoxidase, are involved in the initiation of oxidation of the LDL (oxLDL) [31]. The oxLDL particles are recognized by macrophage scavenger receptors such as scavenger receptor-A (SR-A), CD36 antigen (CD36) and macrophage antigen CD68. Consequently, the oxLDL is rapidly taken up by macrophages that then become enlarged and full of lipids. These cells accumulate in the subendothelial matrix and transform into foam cells, characteristic of the early atherosclerotic lesion (atheroma).

#### Fibrous plaques

Arteries generally consist of three layers, the intima, media and adventitia. The normal media layer contains mostly contractile VSMCs and a few fibroblasts surrounded by their own basement membrane. The major components of the medial extracellular matrix are fibrillar collagen type I and III. In atherosclerosis, the inflammatory response triggers the activated macrophages and T-cells to secrete a number of cytokines and growth factors that promote the change of VSMCs from the quiescent contractile state (differentiation) to the active synthetic state (de-differentiation) [32], the migration from the media to the intima and the production of collagen (fragments of collagen type I, III, and collagen type VIII [33]), elastin and proteoglycan to form a fibrous matrix.

#### Advanced lesions and plaque disruption

The fibrous cap gradually covers the lipids, leading to the death of foam cells and other cell debris that form a necrotic core. The inflammatory response and continuous recruitment of leukocytes and macrophages lead to these lesions and the expansion of their area. The necrotic core represents the secretion of various growth factors (e.g., platelet-derived growth factor [PDGF] and TGF-β), cytokines (e.g., interleukin [IL]-1 and tissue necrotic factor-α [TNF-α]), osteopontin and matrix metalloproteinases (MMPs). Activated T-cells stimulate the production of MMPs, which promote the instability of the lesion and further complicate the inflammatory response. Thinning of the fibrous cap may result from MMPs such as collagenases, elastases, and stromelysins. These MMPs cause the degradation of the matrix, which could lead to hemorrhage from the vasa vasorum or from the lumen of the artery and cause thrombus formation and arterial occlusion.

### Restenosis

Restenosis occurs in patients with atherosclerotic disease undergoing coronary angioplasty with stent replacement. Even with the best medical techniques, restenosis occurs in approximately 30% of patients [34]. Although restenosis and atherosclerosis are recognized as inflammatory processes in response to injury, restenosis has a different pathophysiological appearance than atherosclerosis and has already been considered a developmentally different process [4]. The swelling of diseased vessels by either angioplasty or stent insertion causes endothelial disruption, fragment of the internal elastic lamina, and dissection of the media, often extending into the adventitia. Thus, restenosis after angioplasty or stent insertion is a combination of biological processes, each of which contributes to the final luminal narrowing. Processes observed in animal models and patients include elastic recoil, thrombus, neointima formation and remodeling [35].

#### Elastic recoil

The human coronary artery is highly elastic, with elastin fibers comprising the internal elastin lamina (IEL) and external elastin lamina (EEL). In an eccentric atherosclerotic lesion, the balloon dilatation overstretches the segments of the artery. The elastic recoil occurs within seconds to minutes following balloon dilatation. Over the next days to weeks, the stretched segments become gradually relaxed, leading to a reduction of the luminal diameter. Vasoconstrictors such as serotonin and thromboxane are released by the aggregating platelets that promote vasoconstriction at the site of angioplasty [5].

#### Thrombus

Successful angioplasty usually causes endothelial denudation and induces medial dissection. The consequent exposure of subintimal components, such as collagen, von Willebrand factor, fibronectin, and laminin, causes platelet adherence and aggregation. Many platelets can then become cross-linked by fibrinogen, promoting more platelet aggregation. Platelet aggregation triggers the release of thromboxane A2 and serotonin, which also promotes further adhesion and aggregation. Platelets also secrete a number of mitogens and chemotactic factors for VSMCs, including PDGF and TGF-β, which lead to neointima formation at the site of angioplasty [5].

### *Neointima formation*

Neointima formation, known as intimal hyperplasia, is caused by the proliferation and migration of VSMCs and the accumulation of fibroblasts at the site of injury. Based on the observation of specimens from patients, the migration and proliferation of VSMCs and fibroblasts in the neointimal layer occurs in the weeks to months after angioplasty. Angioplasty induces EC denudation and mechanical stretching of vessels, which lead to the release of various cytokines and growth factors by ECs, inflammatory cells and platelets promoting VSMC proliferation and migration and increasing the synthesis of the collagen, elastin and proteoglycan matrix [36].

#### Remodeling

Remodeling is described as a gradual process of relative changes in vessel size. Remodeling can be classified into positive remodeling (also termed outward/expansive remodeling) and negative remodeling (termed inward/constrictive remodeling). Restenosis may be caused by the negative remodeling of a dilated artery with less neointima formation. In contrast, the positive remodeling of a dilated artery may accumulate large amounts of neointimal tissue. Mintz *et al*. [34] further documented negative remodeling in a series of 209 angioplasty patients and observed that a significant portion of lumen loss was due to vessel constriction rather than neointimal thickening. However, the mechanisms by which negative remodeling can be involved in restenosis remain unclear. The extracellular matrix may be involved in the remodeling of dilated arteries after angioplasty. Angioplasty causes an acute alteration of extracellular matrix synthesis and degradation, resulting in an increase of collagen synthesis and a reduction of MMP activity, reducing matrix degradation.

### Roles of microRNA in vascular cells

#### Endothelial cells

##### *Inflammation*

The pathogenic feature of atherosclerosis is an inflammatory process by which blood vessels respond to injury. Recent studies have reported that miRs are involved in these processes (Table 1). Vessels from swine exhibited decreased expression of miR-10a at athero-susceptible regions of the inner aortic arch and aorta-renal branches. To further demonstrate the role of miR-10a knockdown, the effects of miR-10a knockdown on the endothelial transcriptome were determined in cultured ECs by whole-genome microarray analyses. Bioinformatic analysis identified IκB/NF-κB–mediated inflammation as the main biological processes taking place in miR-10a knockdown cells. Downregulation of miR-10a enhances IκB/NF-κB activation and leads to significant upregulation of inflammatory biomarkers such as MCP-1, VCAM-1, E-selectin, IL-6 and IL-8. This evidence suggests that miR-10a suppresses pro-inflammatory molecules in endothelial phenotypes of the athero-susceptible region in vivo [37]. Through in silico analysis, Harris *et al*. [38] and Wang *et al*. [39] suggested that miR-126 may be a negative regulator of VCAM-1 expression. Overexpression of miR-126 by oligonucleotide transfection led to repression in TNF-α-induced protein expression of VCAM-1 and leukocyte adhesion. Moreover, miR-126 was identified to be involved in the regulation of VCAM-1 at the translational rather than transcriptional level. This result enhances the importance of miR-126 in posttranscriptional gene regulation in ECs. MiR-155 was demonstrated to play an anti-inflammatory role in ECs [40]. Overexpression of miR-155 decreased the adhesion of Jurkat T cells to angiotensin II (Ang II)-stimulated ECs. Endothelin-1 (ET-1) is a potent vasoconstrictive peptide and mitogen that plays multiple roles in the progression of atherosclerosis, vascular inflammation and remodeling. MiR-125a and miR-125b-5p were found to be highly expressed in ECs and are able to suppress the expression of oxLDL-induced ET-1 [41]. Furthermore, miR-132 [42] was also shown to be involved in the inflammatory response of ECs.

**Table 1:** Selected miRs involved in the regulation of vascular endothelial cell function

**Function**	**miRs**	**Targets**	**Reference**
Inflammation	miR-10a	MAP3K7, βTRC	[37]
	miR-125a/125b-5p	prepoET-1	[41]
	miR-126	VCAM-1	[38,39]
	miR-132	AChe	[42]
	miR-155	AT1R, Ets-1	[40]
Angiogenesis	miR-17~92 cluster	ITGA5	[43]
	miR-100	mTOR	[44]
	miR-126	Spred-1, PIK3R2	[39,45-47]
	miR-132	p120RasGAP	[48]
	miR-210	Efn3a	[49]
	miR-221	Cdkn16, PIK3R1	[50]
	miR-222	STST5A	[51]
	miR-424	MEK1, cyclin E1	[52]
	miR-503	Cdc25A, CCNE1	[53]
Migration	miR-21	RhoB	[54]
	miR-150	c-Myc	[55]
	miR-155	Ets-1	[40]
	miR-200a	THBS-1	[56]
	miR-218	Robo1/2, GLCE	[57]
	miR-320	IGF-1	[58]

##### *Angiogenesis*

Angiogenesis is characterized by the formation of new blood vessels from the existing vascular network. Angiogenesis is required in various physiological and pathophysiological conditions such as embryonic development, tissue regeneration, wound healing, tumor growth and atherosclerosis [59]. Cell proliferation and mobility are critical steps for angiogenesis and are strictly controlled by various intracellular signals. MiR profiling of embryonic stem (ES) cell-derived ECs revealed a group of endothelia-enriched miRs, including miR-126, −146, −197 and −625. MiR-126 is most highly enriched in ECs and has been well-characterized as a pro-angiogenic miR. MiR-126 and miR-126* are encoded by intron 7 of the EGF-like domain 7 (Egfl7) gene, which encodes an EC-specific secreted peptide that has been reported to act as a chemoattractant and an inhibitor of smooth muscle cell migration [45]. Knockdown of miR-126 in zebrafish led to a loss of vascular integrity and induced hemorrhage during embryonic development [46]. Targeted deletion of miR-126 in mice led approximately 40% of the miR-126^−^/^−^mice to die embryonically or perinatally. Analysis of embryos obtained from timed matings revealed that miR-126^−^/^−^ embryos were dead or dying, with severe systemic edema, multifocal hemorrhages and ruptured blood vessels throughout embryogenesis [39]. Analysis of gene expression profiles in ECs isolated from miR-126^−^/^−^ and zebrafish morphants demonstrated that miR-126 promoted angiogenesis through VEGF/FGF signaling by targeting its negative regulators Sprouty-related protein-1 (Spred-1) and phosphoinositide-3 kinase regulatory subunit 2 (PIK3R2/p85-b) via the MAPK and PI3K pathways, respectively. The role of hemodynamic forces during embryonic development in the patterning and remodeling of the embryonic circulatory system has been investigated. Nicoli *et al*. [47] further demonstrated that the angiogenic sprouting of blood vessels required the blood flow-induced transcription factor KLF-2, which induced the expression of miR-126 to activate VEGF signaling. This study provided new insights into how ECs respond to flow and integrate developmental signals with miR-126 to promote angiogenesis. Anand *et al*. [48] identified that miR-132 was highly upregulated in human ES during vasculogenesis. Interestingly, miR-132 is also highly expressed in the endothelium of human tumors and hemangiomas, but it is undetectable in the normal endothelium. Overexpression of miR-132 leads to pro-angiogenic signals, proliferation and Ras activity via suppression of p120RasGAP in ECs. Furthermore, selective delivery of anti–miR-132 through α_v_β_3_ integrin-targeted nanoparticles to the tumor endothelium of mice reduced the tumor burden and angiogenesis.

Dicer is an important RNase III enzyme for miR maturation. Suarez *et al*. [60] clarified that the knockdown of Dicer in ECs altered the expression of angiogenic regulators such as Tie-2, endothelial nitric oxide synthase (eNOS) and IL-8. Knockdown of Dicer in ECs results in a reduction of proliferation via cell cycle delay from the G1 into the S phase, along with impairment of cord formation. The miR-17~92 cluster (encoding miR-17, -18a, -19a/b-1, -20a, and -92a) is overexpressed in several tumor cells and in the regulation of angiogenesis. Bonauer *et al*. [43] demonstrated that miR-92a was highly expressed in ECs and exhibited anti-angiogenic activity by targeting several endothelial functional genes, including integrin subunit α_5_ and α_v_, sphingosine-1-phosphate receptor-1 (SIP-1), and mitogen-activated protein kinase (MAPK) kinase-4 (MKK-4). These endothelial functional genes mediate the cell-matrix interaction, cell migration and angiogenesis. Furthermore, the mouse hind limb ischemia model and myocardial infarction model demonstrated that antagomir-92a led to enhanced growth of blood vessels and functional recovery of damaged tissue. Moreover, miR-210 [49], miR-221 [50], miR-222 [51], miR-100 [44], miR-424 [52] and miR-503 [53] have also been shown to play critical roles in the modulation of angiogenesis (Table 1).

##### *Migration*

Endothelial migration is an important property of angiogenesis. This motile ability is regulated by growth factors, chemotactic factors and mechanical forces. These factors trigger several signaling networks that converge on cytoskeleton remodeling in migrating cells. In recent studies, several miRs were reported to be involved in the regulation of migration through impaired cytoskeleton remodeling related to transcription factors and signaling molecules (Table 1). An interesting article reported by Zhang *et al*. [55] demonstrated that secreted monocytic miR-150, which is packaged by the microvesicles (MVs), could enter and be delivered into human microvascular ECs (HMECs), thus enhancing cell migration and decreasing c-myc expression. These studies further revealed that blood cells and cultured THP-1 cells are able to selectively package other immune-related miRs such as miR-146a and miR-181a into MVs in response to various stimuli. In addition, miR-200a has been shown to promote EC migration via the repression of thrombrospodin-1 (THBS-1) [56]. The important miR-155 has multiple functions in ECs, not only in the regulation of inflammation, but also in the inhibition of EC migration in response to Ang II [40]. Ets-1 is an important endothelial transcription factor that robustly regulates endothelial inflammation, angiogenesis and vascular remodeling. Bioinformatic and luciferase assays demonstrate that Ets-1 can be directly targeted by miR-155 in two potential target sites of the 3’-UTR region. Slit-Robo signaling controls the angiogenesis and contributes to the development of the vascular network. Small *et al*. [57] demonstrated that miR-218 was expressed from the slit2 and slit3 genes, resulting in further direct repression of the expression of Robo1, Robo2, and glucuronyl C5-epimerase (GLCE), resulting in a reduction of EC migration. This intact miR-218-Slit-Robo regulatory network is necessary for the vascularization of the retina. MiRs have been reported to decrease EC migration, including miR-21 [54] and miR-320 [58] via repression of RhoB and insulin-like growth factor-1 (IGF-1), respectively.

#### Macrophages/monocytes

Monocytic differentiation and oxLDL uptake are critical processes in atherosclerosis. Wang *et al*. [61] integrated microarray data and a bioinformatic database to reveal the correlations between miR and target mRNA in the TPA-induced differentiation of U937 cells. Fontana *et al*. [62] demonstrated the role of miR-17-5p-20a-106a in one monocyte lineage from the cord blood CD34^+^ hematopoietic progenitor cells (HPCs). MiR-17-5p–20a–106a suppresses AML1 protein expression, leading to the downregulation of the M-CSF receptor (M-CSFR) and the inhibition of monocytopoiesis. In contrast, using the same model cell type, Rosa *et al*. [63] revealed that miR-424 promoted monocytic differentiation through the repression of NFI-A, the transcription factor used to regulate monocytic differentiation. An Agilent miR array revealed that miR-155, −222, -424, and −503 are involved in monocytic differentiation through cell cycle arrest and apoptosis [64]. In addition to these miRs, miR-155 is also implicated in the regulation of monocyte-derived dendritic cells [65], macrophage inflammatory responses [66] and uptake of oxLDL. Huang *et al*. [67] demonstrated the miR-155 could reduce the lipid uptake in oxLDL-stimulated and PMA-differentiated THP-1 cells. MiR-125a-5p was shown to decrease lipid uptake and the secretion of inflammatory cytokines, including IL-2, IL-6, TNF-α and TGF-β, in oxLDL-stimulated human primary monocytes via the repression of oxysteral binding protein like-9 (ORP9) [68]. MiR-33 has been reported to play a role in sterol transport [69,70]. MiR-33 is an intronic miR that localizes within the gene encoding sterol-regulatory element–binding factor–2 (SREBF-2) and acts as a transcriptional regulator of cholesterol synthesis to modulate the expression of genes related to cholesterol transport. MiR target prediction algorithms and overexpression of miR-33 in mouse macrophages identified the adenosine triphosphate–binding cassette transporter (ABCA-1) as a miR-33 target gene. Antagonism of endogenous miR-33 increased ABCA1 protein and cholesterol efflux to apolipoprotein A1 in both murine and human macrophages (Table 2).

**Table 2:** Selected miRs involved in the regulation of macrophage/monocyte function

**miRs**	**Function**	**Targets**	**Reference**
miR-17-5p-200-106a	Monocytopoiesis	AML1	[62]
miR-33	Sterol transport	ABCA-1	[69,70]
miR-125a-5p	Lipid uptake	ORP9	[68]
miR-155	Differentiation	TAB2, MyD88	[65-67]
	Inflammation		
	Uptake of oxLDL		
miR-424	Differentiation	NFl-A	[63,64]

#### Smooth muscle cells

Neointima formation is commonly attributed to VSMC proliferation. Several reports have demonstrated the involvement of miRs in the mediation of VSMC proliferation and migration (Table 3). In rat balloon-injured carotid arteries and cultured rat VSMCs, miR-21 [71], miR-221 [72] and miR-222 were shown to play roles in the regulation of VSMC proliferation through phosphatase and tensin homology (PTEN), B-cell lymphoma 2 (Bcl-2) and p27(Kip1), p57(Kip2), respectively. PTEN and Bcl-2 have been reported to serve as important molecules associated with VSMC proliferation and apoptosis. p27(kip1) and p57(kip2) are critical molecules involved in cell cycle regulation and were demonstrated to be negative regulators in VSMC proliferation [73]. In general, miR-146a is known to serve an anti-inflammatory function in various cells (as mentioned above). Sun *et al*. [74] further substantiated that miR-146a directly targets Krupple-like factor-4 (KLF-4) and demonstrated its important role in promoting VSMC proliferation in cultured rat VSMCs and vascular neointimal hyperplasia. Interestingly, miR-146a and KLF-4 formed a feedback loop regulating each other’s expression. KLF-4 inhibited miR-146a at the transcriptional level, while miR-146a inhibited the expression of KLF-4 by targeting the 3’-UTR region of KLF-4. Another member of the KLF family, KLF-5, promoted the transcription of miR-146a and acted as a competitor with KLF-4. These molecules form a regulatory circuitry to precisely modulate the proliferation of VSMCs. Wu *et al*. [75] found that miR-130a correlated with vascular remodeling in spontaneously hypertensive rats (SHRs). MiR-130a was up-regulated in the thoracic aorta and mesenteric arteries of SHRs. In addition, the mRNA expression and protein level of growth arrest-specific homeobox (GAX) were downregulated by miR-130a. MiR-130a mimics at 25 or 50 nmol/l significantly promoted the proliferation of VSMCs.

**Table 3:** Selected miRs involved in the regulation of VSMC function

**Function**	**miRs**	**Targets**	**Reference**
Increase proliferation	miR-21	PTEN, Bcl-2	[71]
	miR-130a	GAX	[75]
	miR-146a	KLF-4	[74]
	miR-221/222	p27, p57	[72,73,76]
Decrease proliferation	miR-26	SMAD	[77]
	miR-133	Sp-1	[78]
	miR-143/145	KLF-5	[79,80]

Some miRs were found to take part in the repression of VSMC proliferation. The miR-143/145 cluster is abundantly expressed in the normal vessel walls. Interestingly, miR-143/145 is dramatically downregulated in injured carotid arteries after angioplasty [79,80]. MiR-143 is highly conserved and lies within 1.7 kilobases (kb) of another miR145 on the mouse chromosome 18. Both miRs are downregulated in various cancer cell lines [81]. Cheng *et al*. [80] further demonstrated that miR-145 is a critical modulator for VSMC differentiation through its target gene KLF-5. The expression of VSMC differentiation marker genes such as SM α-actin, calponin and SM-MHC were increased at the gene and protein levels using a miR-145 mimic oligonucleotide. In contrast, overexpression of KLF-5 reduced the gene expression of SM α-actin. These results provide evidence of a correlation between miR-145 and KLF-5 in VSMC differentiation. MiR-26a was selected from growth-arrested human aortic SMCs by an miR array [77]. This profile revealed that miR-26a was significantly upregulated in differentiated VSMCs through a decrease of SMAD activity. In addition, miR-26a was dramatically downregulated in two murine AAA development models, abdominal aortic aneurysms (AAAs) and ApoE^−^/^−^/AngII aneurysm. MiR-133 is robustly expressed in VSMCs in vitro and in vivo [78]. In serum-starved synchronized adult rat carotid VSMCs, miR-133 was abundant and indirectly regulated VSMC marker genes and proteins through the Sp-1 transcription factor.

### Roles of microRNA in atherosclerosis

Blood vessels are constantly subjected to various hemodynamic forces, including hydrostatic pressure, cyclic stretch and fluid shear stress. As the monolayer is in direct contact with flowing blood, vascular ECs are constantly exposed to blood flow-induced shear stress. Extensive evidence has shown that hemodynamic forces may play prominent roles in the development of vessel maturation, physiology and pathophysiology. Atherosclerosis occurs preferentially in arterial branches and curvatures where the shear stress is low and dynamic [2], and the initial step is attributed to EC dysfunction. Oscillatory shear stress (OSS) induces the expression of miR-21 at the transcriptional level in cultured ECs and eventually leads to an inflammatory response through peroxisome proliferators-activated receptor-α by 3'-UTR targeting [82]. Wu *et al*. [83] demonstrated that pulsatile shear stress (PSS) downregulated but OSS upregulated the expression of miR-92a in ECs. Previous studies have shown that KLF-2 was significantly upregulated by atheroprotective shear flow such as PSS and laminar shear stress. Bioinfomatic analysis demonstrated that KLF-2 serves as a target gene for miR-92a, and its gene and protein levels were downregulated by OSS-stimulated ECs. In addition, the KLF-2 regulated-genes such as eNOS and thrombomodulin (TM) were repressed by overexpression of miR-92a in ECs. This study provides a new concept for the regulatory circuitry of the responses of KLF-2 and miRs to atheroprotective shear flow. MiR-663 [84], miR-19a and miR-23b [85,86] have also been studied and shown to be regulated by shear stress and involved in the modulation of EC inflammation and proliferation, respectively.

The functions of various miRs and their involvement in biological processes have been identified in various cultured cells or animal models. The expression profiles of circulating miRs [87] and peripheral blood mononuclear cells (PBMCs) [88-91] in patients with cardiovascular diseases have been extensively studied. Unfortunately, the involvement of miRs in human atherosclerotic plaques has received little attention. Raitoharju *et al*. [92] were the first to investigate the miR/mRNA expression profiles in human atherosclerotic plaques from peripheral arteries (carotid, femoral, and the aorta) in comparison to non-atherosclerotic left internal thoracic arteries (LITA), and they elucidated the relationship between miR/mRNA expression profiles and biological processes in atherosclerosis. They found that miR-21, -34a, -146a, -146b-5p, and −210 were expressed at significant levels, and numerous predicted targets of these miRs were downregulated in human atherosclerotic plaques. The combination of miR/mRNA profiles and bioinformatic analysis showed that nine KEGG pathways were enriched with predicted targets, including immunodeficiency, metabolism, p53 and cell proliferation signaling pathways. Interestingly, among these pathways, cancer-related pathways are significantly upregulated. In contrast, VSMC contraction and purine metabolism were downregulated in human atherosclerotic plaques compared to LITAs. MiR-34a was identified as a new target for atherosclerotic pathogenesis due to its function in apoptosis and cell cycle arrest, its modulation of the p53 signaling pathway, and its target genes related to VSMC proliferation and cholesterol metabolism. Taken together, these links strongly support the connection of miR-34a to cardiovascular diseases. MiR-146a is highly expressed in both human atherosclerotic plaques and PBMCs [88,89] in patients with cardiovascular diseases It was previously shown that the miR-146 family (miR-146a/b) regulated downstream toll-like receptor 4 (TLR4) signaling, IL-1 receptor associated kinase-1 (IRAK1) and TNF-receptor associated factor-6 (TRAF6) through a negative-feedback regulation loop. IRAK and TRAF6 activated the downstream transcription factors NF-κB and AP-1 and then upregulated the TLR4-mediated immune response. Elevated miR-146 expression was shown to act in an NF-κB-dependent manner by utilizing an LPS (lipopolysaccharide)-stimulated human monocytic cell line [93].

Recent studies demonstrated that miRs can be transferred through the gap junction or secreted between cells [94-96]. Surprisingly, miRs are present in serum or plasma in a remarkably stable form that even resists repetitive freezing/thawing cycles and are protected against RNases. Fichtlscherer *et al*. [87] performed a miR profile using RNA isolated from 8 healthy volunteers and 8 patients with stable coronary artery disease. The circulating levels of angiogenesis-related miR-126 and miR-92a, the inflammation-associated miR-155, VSMC–enriched miR-145 and miR-17 are significantly reduced in patients with coronary artery disease compared with healthy controls. In contrast, cardiac muscle–enriched miRs, miR-133a and miR-208a levels were shown to be elevated in patients with coronary artery disease. The exact mechanisms of reduction of circulating miRs remain unclear. Supposedly, the activity of ECs may contribute to the lower levels of circulating miRs. Another implication may be that circulating miRs are taken up into atherosclerotic lesions, leading to a reduction of circulating miRs in blood. Overall, this article raises the potential role of circulating miRs as biomarkers for diagnosis of cardiovascular diseases.

### Roles of microRNA in restenosis

Rat carotid artery balloon injury is a common animal model to study restenosis [36]. Ji *et al*. [71] were the first to determine the miR profile in the rat carotid artery after balloon injury utilizing an miR array. Aberrant overexpression of miR-21 was determined at a significant level in neointimal lesions. The miR-21 gene is located on the plus strand of chromosome 17q23.2 within the coding gene TMEM49 (also known as vacuole membrane protein). This gene was first described as an oncomir due to its abundant expression in various cancers [97]. MiR-21 is involved in the promotion of VSMC proliferation and anti-apoptosis by directly targeting PTEN and PDCD4 [98], respectively. In addition, Liu *et al*. [72] and Davis *et al*. [76] clarified the role of miR-221 and miR-222 in VSMC proliferation and neointimal hyperplasia. MiR-221 and miR-222 are encoded by a gene cluster on the X chromosome, they share the same seed and appear to have identical target genes and similar functions. Both miRs are significantly mediated by PDGF-BB and serum treatment in cultured VSMCs. Liu *et al*. [72] further demonstrated that expression of miR-221 and miR-222 were upregulated in balloon-injured rat carotid arteries and their target genes, p27(Kip1) and p57(Kip2), were downregulated. Downregulation of miR-221 and miR-222 reduced the proliferation of VSMCs and neointima formation in the rat carotid artery after angioplasty.

Recently, several studies demonstrated the role of the miR-143/miR-145 cluster in VSMC differentiation and vascular disease [79,80,99-101]. Cordes *et al*. [79] first revealed the distribution of miR-143/miR-145 during embryonic development. Postnatally, the transcript levels of the miR-143/miR-145 cluster are high in smooth muscle of the aorta, pulmonary artery and coronary vessels but undetectable in the ventricular myocardium. Furthermore, miR-143 and miR-145 cooperatively target a network of transcriptional factors including Elk-1 (ELK1 is a member of the ETS oncogene family), KLF-4 and myocardin to promote differentiation and repress the proliferation of VSMCs. MiR-143/miR-145 knockout (KO) mice were also established in advance to clarify the maintenance of the contractile phenotype of VSMCs [99-101]. Elia *et al*. [100] showed that the aorta of apolipoprotein E (ApoE) KO mice, in which vascular damage is enhanced by a hypercholesterol diet, exhibits markedly decreased constitutive levels of miR-143 and miR-145. Albinsson *et al*. [102] generated Dicer KO mice and found late embryonic lethality at embryonic day 16 to 17 associated with extensive internal hemorrhage. Expression of miRs, including miR−21, −221, -145 and VSMC-specific marker genes are significantly reduced in SMC-Dicer KO vessels. Interestingly, overexpression of miR-145 rescued SMC-specific mRNA and protein expression in dicer KO SMC by miR-145 mimics. This finding indicates that an additional miR-dependent mechanism is necessary during VSMC development rather than Dicer because the loss of Dicer in mice is lethal. These studies demonstrated the important role of miR-145 in VSMC differentiation and vascular disease.

### Summary and conclusion

Atherosclerosis is a widespread condition with high morbidity and mortality in both developed and developing countries. Its complications, including unstable coronary syndromes, myocardial infarction and stroke, usually cause high mortality in patients. Several medications and surgical procedures have been used for clinical therapy. Patients with atherosclerotic disease are usually treated by angioplasty with stent replacement. However, restenosis is commonly observed in angioplasty patients. Both pathologies are underlined by complicated pathophysiological processes, and extensive studies on cellular mechanisms have been well established to seek opportunities for clinical therapy. MiRs are a novel class of gene regulators, and their important roles and functions in vascular biology have been demonstrated in over 400 reports. This review summarizes the current understanding of the roles of miRs in atherosclerosis and restenosis. ECs, VSMCs and blood cells contribute to both vascular pathologies. Each cell type has a specific role in these two conditions, with ECs exhibiting an inflammatory response, angiogenesis and migration; VSMCs undergoing differentiation and proliferation; and blood cells modulating oxLDL uptake and lipid metabolism. Hence, we focus on the different features of each type of cell to elucidate how miRs modulate these cellular functions. We discussed the significant changes in miR expression profiles that occur in human specimens with atherosclerosis and animal models with angioplasty. These profiles led to new insight into the potential clinical applications of miRs and emphasize the importance of miRs in the pathogenic processes of vascular diseases. Interestingly, some miRs are altered in vitro and in vivo studies, for example, miR−126, −17~92a, −145, −21 and −146a. Some miRs can only be expressed in specific tissues or cells with a special status. The EC-specific miR-126 and VSMC-specific miR-145 are usually enriched in blood vessels during embryonic development and in mature vessels. Supposedly, these miRs are involved in the maintenance of homeostasis or development of blood vessels. MiR-21 and miR-221/222 have been investigated as promoters of the proliferation of VSMCs via negative modulation of cell cycle regulation as well as of PTEN and p27. MiR-21 is also referred to as an oncomir due to its high expression levels in various cancer cell lines. This implies that these miRs contribute to vascular pathogenesis. Some miRs are expressed in multiple cells, such as miR-146a and miR-155, which are expressed in both ECs and blood cells to induce a cellular inflammatory response and protect blood vessels, respectively. This indicates that miRs may have great potential as therapeutics. Interestingly, recent advances have enabled the identification of miRs released into circulating blood from injured tissue or highly expressed in patients with cardiovascular diseases. This implies that circulating miRs and tissue/cell-specific miRs are potential biomarkers for clinical diagnosis in patients with cardiovascular diseases. The overall body of evidence shows that miRs have emerged as a new layer of complexity in vascular diseases and may represent novel biomarkers and new therapeutic targets for cardiovascular diseases.

## Abbreviations

3’-UTRs, 3’-untranslated regions; ABCA-1 transporter, adenosine triphosphate–binding cassette; Ang II, angiotensin II; ApoE, apolipoprotein E; Ago2, argonaute 2; Bcl-2, B-cell lymphoma 2; BMP, bone morphogenetic protein; CD36, CD36 antigen; 8DGCR, DiGeorge critical region 8; Egfl7, EGF-like domain 7; eNOS, endothelial nitric oxide synthase; ET-1, endothelin-1; XPO5, exportin-5; EEL, external elastin lamina; GLCE, glucuronyl C5-epimerase; GAX, growth arrest-specific homeobox; HPCs, hematopoietic progenitor cells; HMECs, human microvascular ECs; IRAK1, IL-1 receptor associated kinase-1; IGF-1, insulin-like growth factor-1; ICAM-1, intercellular adhesion molecule-1; IL-1, interleukin; IEL, internal elastin lamina; KO, knockout; KLF-4, krupple-like factor-4; LITA, left internal thoracic arteries; LPS, lipopolysaccharide; LDL, low-density lipoprotein; M-CSF, macrophage colony stimulating factor; MMPs, matrix metalloproteinases; M-CSFR, M-CSF receptor; mRNA, messenger RNA; miRs, microRNAs; MVs, microvesicles; miRISC, miR-induced silencing complex; MKK-4, mitogen-activated protein kinase kinase-4; MCP-1, monocyte chemotactic factor-1; OSS, oscillatory shear stress; oxLDL, oxidation of the LDL; ORP9, oxysteral binding protein like-9; Pasha, partner of Drosha; PTCA, percutaneous transluminal coronary angioplasty; PBMCs, peripheral blood mononuclear cells; PTEN, phosphatase and tensin homology; PIK3R2/p85-b, phosphoinositide-3 kinase regulatory subunit 2; PDGF, platelet-derived growth factor; pol II, polymerase II; pol III, polymerase III; pri-miRs, primary miRs; PSS, pulsatile shear stress; ROS, reactive oxygen species; RNASEN, RNase III enzyme Drosha; SR-A, scavenger receptor-A; SR, scavenger receptors; sPLA2, secretory phospholipase-2; SIP-1, sphingosine-1-phosphate receptor-1; Spred-1, sprouty-related protein-1; SREBF-2, sterol-regulatory element–binding factor–2; TRBP, Tar RNA binding protein; TM, thrombomodulin; THBS-1, thrombrospodin-1; TNF-α, tissue necrotic factor-α; TRAF6, TNF-receptor associated factor-6; TLR4, toll-like receptor 4; TGF-β, transforming growth factor-β; VCAM-1, vascular cell adhesion molecule-1; ECs, vascular endothelial cells; VSMCs, vascular smooth muscle cells.

## Competing interests

The authors declare that they have no competing interests.

## Authors’ contributions

LJC and JJC wrote the manuscript. SHL, YTY, and SCL assisted with the revision of English grammar and style. All authors discussed the content and approved the final version of manuscript.
